# Case Report: Neonaticide, infanticide, and filicide — psychopathological aspects and forensic implications

**DOI:** 10.3389/fpsyt.2026.1824900

**Published:** 2026-10-19

**Authors:** Renata de Souza Rosa Lima, Julia de Bulhões Carvalho Schechtman, Antonio Egidio Nardi, Alexandre Martins Valença

**Affiliations:** 1 Institute of Psychiatry (IPUB), Federal University of Rio de Janeiro (UFRJ), Rio de Janeiro, Brazil; 2 Department of Psychiatry and Mental Health, Fluminense Federal University (UFF), Rio de Janeiro, Brazil

**Keywords:** depression, filicide, infanticide, mental illness, neonaticide, psychosis, schizophrenia

## Abstract

This article offers an in-depth analysis of child homicide committed by parents, using two clinical cases to illustrate the complexity of the issue. A variety of social, psychological, and educational factors contribute to this serious fatality. Mental disorders such as mood disorders and psychotic conditions often play a significant role. The article highlights the critical need for mental health services to address issues such as treatment disengagement and non-adherence, which are frequently precursors to violent behavior in individuals with severe mental illnesses. Additionally, providing support to socially vulnerable pregnant women, even those without mental disorders, is critically important. To help mitigate risks and prevent family tragedies the assessment of the presence of psychiatric symptoms during the postpartum period by psychiatrists, obstetricians, and pediatricians is essential.

## 1 Introduction

Filicide is a rare but clinically, socially, and legally significant form of homicide. The risk of becoming a homicide victim is particularly high during the first year of life, when an individual is approximately four times more likely to be killed than at any other period (1). Infant homicide rates have been estimated at 4.5 per 100,000 live births in England and 8 per 100,000 in the United States (1). Considerable international variation has been reported: a systematic review found neonaticide rates ranging from 0.07 per 100,000 births in Finland to 8.5 in Austria, with rates exceeding 5 per 100,000 in Lithuania and Estonia and reaching 27.7 per 100,000 in Tanzania (2).

The terminology used to describe parental child homicide depends partly on the age of the victim. In the international literature, neonaticide generally refers to the killing of a child within the first 24 hours of life, infanticide to the killing of an infant from one day to one year of age, and filicide more broadly to the killing of a child by a parent (3). These distinctions are clinically relevant because the circumstances, psychiatric correlates, and psychosocial characteristics associated with the killing of a newborn may differ substantially from those observed when parents kill older children.

Sociocultural conditions may also influence both the occurrence and interpretation of these offenses. Sexual education, access to effective contraception and legal abortion, women’s social and financial autonomy, stigma surrounding pregnancy, and family and community support have been proposed as factors contributing to international differences in neonaticide rates (4). In some African contexts, traditional beliefs involving witchcraft, spirit possession, twins, or children with albinism have also been identified as relevant to understanding neonaticide and other forms of filicide (4). Thus, filicide cannot be understood exclusively through psychiatric diagnosis; it occurs at the intersection of psychopathology, psychosocial vulnerability, family relationships, cultural context, and legal frameworks.

This complexity is particularly important in Brazil, where the distinction between homicide and infanticide has direct legal consequences. Under the Brazilian Penal Code (5), a mother who kills her child during or shortly after childbirth may be charged with infanticide when the offense is committed under the “influence of the puerperal state.” Infanticide carries a substantially lower penalty of two to six years of imprisonment, compared with six to twenty years for homicide. When the influence of the puerperal state is not established, the offense may instead be classified as homicide (5). Consequently, determining the relationship between childbirth, mental state, psychiatric disorder, psychosocial circumstances, and criminal behavior represents a particularly important challenge in forensic psychiatric assessment.

The present article examines this problem through two cases of women who committed filicide and subsequently underwent forensic psychiatric evaluation. To provide a framework for interpreting these cases, the conceptual analysis focuses on three interrelated dimensions: (1) the heterogeneity and classification of filicide; (2) the different roles of psychiatric disorders and psychosocial vulnerability across neonaticide, infanticide, and filicide; and (3) the relationship between filicide, suicidality, and severe psychopathology. This framework is intended to move beyond a single-cause explanation and to clarify the factors that should be considered when evaluating maternal filicide in forensic practice.

The study was approved by the Ethics Committee of the Institute of Psychiatry at the Federal University of Rio de Janeiro, and the patients provided informed consent to participate.

## 2 Conceptual analysis

### 2.1 Filicide as a heterogeneous phenomenon: from motive-based to empirically derived classifications

One of the major difficulties in interpreting filicide is that it does not represent a single clinical or criminological phenomenon. Parents may kill their children under markedly different psychiatric, interpersonal, and social circumstances. Early attempts to organize this heterogeneity focused primarily on the presumed motivation underlying the offense.

Resnick’s classic review of psychiatric literature on child homicide between 1751 and 1967 examined 131 cases and proposed five categories of filicide (6). Although the study predated standardized contemporary psychiatric diagnostic criteria, its classification remains influential because it illustrates the diversity of pathways leading to parental child homicide.

The first category, altruistic filicide, describes cases in which the parent believes that death will protect the child from actual or imagined suffering. It includes situations in which the parent perceives an inescapable threat to the child or kills the child in association with a planned parental suicide. Severe depression, including psychotic depression with delusions or hallucinations, and schizophrenia have frequently been associated with this category (7).

The second category, acutely psychotic filicide, refers to killings committed under the influence of severe psychotic symptoms without an otherwise comprehensible external motive such as revenge, punishment, or material gain. The third, unwanted-child filicide, occurs when the child was never wanted or is no longer desired and has historically been associated with younger and unmarried mothers, unwanted or concealed pregnancies, and psychosocial vulnerability rather than severe mental illness (1, 8, 9).

The fourth category, accidental filicide, encompasses deaths resulting from severe abuse or neglect in which death itself was not intended, including cases historically associated with battered child syndrome. Finally, spousal-revenge filicide involves killing the child as a means of retaliating against or inflicting suffering on the other parent. Such cases have been associated with severely dysfunctional relationships, emotional instability, self-harm, and personality pathology (7, 10).

More recent research has shifted from classifications based primarily on presumed motives toward empirically derived perpetrator profiles. Using latent class analysis of filicide cases from Austria and Finland, Putkonen et al. (2016) (11) identified five groups: Homicidal-Suicidal Fathers; Violent, Impulsive Parents; Single, Sober Parents; Prosocial, Psychotic Parents; and Infanticidal Mothers. These groups differed in sex, psychiatric history, criminal background, family circumstances, and characteristics of the offense. Such findings reinforce an important conceptual point: filicide is better understood as a heterogeneous outcome arising through different pathways than as the manifestation of a single psychiatric condition or motive.

This distinction provides the first component of the framework used to interpret the cases presented in this article: the forensic assessment should identify the pathway and circumstances surrounding the offense rather than assume that all maternal filicides share the same psychopathological mechanism.

### 2.2 Neonaticide and the importance of psychosocial vulnerability

The relationship between mental disorder and maternal child homicide varies substantially according to the age of the victim. This is particularly evident in neonaticide. Contrary to the assumption that the killing of a newborn is necessarily associated with severe postpartum mental illness, several studies have identified a profile characterized more strongly by psychosocial vulnerability, concealed or unwanted pregnancy, younger age, and unmarried status.

An early English study examined 89 women admitted to prison hospital wards between 1970 and 1975 after killing 109 children (9). Only 24 were considered mentally ill, and only 14 were diagnosed with psychotic disorders. Importantly, none of the women who committed neonaticide were considered mentally ill. Psychiatric disorders were more strongly associated with the killing of children aged one year or older. Neonaticide, by contrast, was associated with younger age and unmarried status. The authors concluded that puerperal psychosis was a relatively uncommon explanation for neonaticide (9).

A Brazilian retrospective study of 53 women accused of killing children within the first 24 hours of life in Rio de Janeiro between 1900 and 1995 similarly demonstrated the importance of social circumstances (12). Most women were unmarried migrant domestic workers with limited education. Eleven were referred for forensic psychiatric assessment, nine were actually evaluated, and only four reported abnormal mental states during the offense. One was diagnosed with intellectual disability, whereas the remaining women were considered mentally normal (12).

More recent evidence supports this distinction. The systematic review by Milia and Noonan (13) found that psychosis was less prominent in neonaticide than in child killings occurring after the neonatal period. Neonaticide was more commonly associated with pregnancy denial and psychosocial vulnerability, whereas infanticide and filicide involving older children showed stronger associations with severe depressive episodes and prolonged suicidal ideation.

Taken together, these findings caution against automatically equating childbirth-related child homicide with puerperal psychosis. They also have particular forensic relevance in jurisdictions such as Brazil, where the legal concept of the “puerperal state” can influence whether the offense is classified as infanticide or homicide. The temporal proximity of the offense to childbirth therefore cannot, by itself, establish the presence of a severe mental disorder or its causal relationship to the crime.

### 2.3 Filicide of older children: severe mental disorders and psychopathology

A different clinical pattern becomes more prominent when the victim is beyond the neonatal period. Across forensic samples, filicide involving older children has repeatedly been associated with severe mood disorders, psychotic disorders, delusions, hallucinations, and suicidal behavior.

A Turkish case series examining 85 filicidal parents and 96 victims between 1995 and 2000 illustrates this pattern (14). Forty-eight percent of perpetrators were men and 52% were women. Maternal perpetrators predominated among victims younger than one year, whereas fathers predominated among victims aged 12–18 years. Forensic psychiatric assessment concluded that 41 perpetrators (48%) were legally insane; among these, 25 had schizophrenia, nine major depression, and seven intellectual disability (14).

The importance of severe psychopathology is even clearer in samples selected through the insanity defense. In a retrospective study of 39 filicidal women found not guilty by reason of insanity, 82% had a psychotic or mood disorder (15). At the time of the offense, 49% were depressed, 13% manic, 59% experienced auditory hallucinations—particularly command hallucinations—and 74% had delusions, frequently involving the child. Approximately half attempted suicide immediately after the filicide (15).

In this sample, 54% of the offenses were classified as altruistically motivated, and 85% of these altruistic motivations were psychotic in nature (15). This observation is conceptually important because the term “altruistic” describes the parent’s perceived motivation rather than the absence of psychopathology. A parent may believe that killing a child is protective or beneficial precisely because severe depression, delusions, or hallucinations have profoundly distorted the perception of the child’s circumstances.

A forensic-center study in New York provides additional evidence (16). Among 57 women who committed or attempted filicide, 63% had schizophrenia-spectrum disorders and 30% had mood-spectrum disorders, including major depression, psychotic depression, postpartum depression, or bipolar disorder. Compared with the profile commonly described in neonaticide, these women tended to be older and to kill older children. Severe depression, psychosis, and self-directed violence were prominent features (16).

Population-level findings indicate that psychiatric morbidity is important but not universal. In an English study of 2,660 homicide offenders, 112 had killed a child (17). Biological fathers accounted for 50% of perpetrators and biological mothers for 31%. Thirty-eight percent had a documented psychiatric diagnosis before the homicide and 23% had previously been diagnosed with a severe mental disorder. Psychiatric disorders were particularly prevalent among female perpetrators (17).

These studies collectively suggest a clinically meaningful gradient: psychosocial and pregnancy-related factors appear particularly important in many cases of neonaticide, whereas severe affective and psychotic disorders become increasingly relevant in maternal filicide involving older children. This distinction should not be interpreted deterministically, since substantial overlap exists between groups, but it provides a more useful framework than treating all forms of maternal child homicide as clinically equivalent.

### 2.4 Filicide-suicide and the intersection between psychopathology and perceived altruism

Suicidality represents another important dimension in the conceptual understanding of filicide. Reported rates of suicide following filicide range from approximately 16–29% among mothers and 40–60% among fathers (15). In some cases, the child homicide and parental suicide appear to constitute components of a single psychopathological process rather than independent behaviors.

Resnick observed that some parents classified as altruistic or acutely psychotic appeared to experience a reduction in psychological tension after killing the child (6). This phenomenon has been proposed as one possible explanation for why some individuals who initially intended to die by suicide after killing their children ultimately survive.

A retrospective study of 30 filicide-suicide cases occurring between 1958 and 2002 included 10 mothers and 20 fathers who killed a total of 51 children and adolescents (18). Evidence of depressive symptoms was present in 57% and psychosis in 27%. Most cases were classified as altruistically motivated. In some, psychotic beliefs concerning the child’s current or anticipated suffering contributed directly to the offense (18).

These findings are consistent with the study of women found not guilty by reason of insanity, in which approximately half attempted suicide immediately after killing their children (15). Together, the evidence suggests that the combination of severe depression, suicidal intent, psychotic beliefs concerning the child, and perceived altruistic motivation constitutes a particularly important pathway to filicide.

This pathway is clinically and forensically distinct from neonaticide associated primarily with unwanted or concealed pregnancy and from filicide occurring in the context of interpersonal retaliation, abuse, or impulsive violence.

### 2.5 Gender, family context, and an integrative framework for forensic interpretation

Psychiatric symptoms alone do not fully explain filicide. Family conflict, domestic violence, socioeconomic adversity, substance use, social isolation, unwanted pregnancy, and cultural factors may interact with individual psychopathology and influence both the occurrence and form of the offense (19).

Recent evidence also highlights important gender differences. Cuadros and Lorente (2026) (20) found that female perpetrators tend to be younger and that their offenses are more frequently associated with unwanted pregnancy, psychiatric disorders, social deprivation, and lack of support. Neonaticide and infanticide occur predominantly among mothers. Male perpetrators tend to be older and are more frequently associated with intimate-partner conflict, jealousy, revenge, and impulsivity. Filicide more broadly often occurs within contexts of domestic violence and family dysfunction. The review also identified gender differences in legal and institutional responses and in media representations of filicidal mothers and fathers (20).

These observations reinforce the need for an integrative rather than diagnosis-centered approach. Maternal filicide should be conceptualized as a heterogeneous behavior arising from different combinations of psychiatric, psychosocial, interpersonal, cultural, and situational factors. The age of the child, timing in relation to childbirth, presence or absence of psychosis or severe mood disorder, suicidal intent, pregnancy circumstances, family conflict, and the parent’s perceived motivation all contribute to distinguishing these pathways.

## 3 Case reports

### 3.1 Case 1

A., a 28-year-old single brazilian woman who was unemployed at the time of the forensic evaluation. She was accused of killing her newborn son in 2015 and was charged under Article 121 of the Brazilian Penal Code (21).

The patient reported growing up in a socially vulnerable environment and denied any family history of psychiatric disorders or neurological diseases. She denied previous psychiatric treatment, suicide attempts, head trauma, epilepsy, or use of alcohol or illicit drugs. At the age of 21, she had been involved in a motorcycle accident that resulted only in a lower-limb injury, without neurological sequelae.

Regarding the pregnancy, she reported that it was unplanned and remained concealed from most relatives throughout gestation. According to her account, she intentionally hid the pregnancy because she feared family and social reactions and because the child’s father refused to acknowledge paternity after being informed of the pregnancy. She stated that he became physically aggressive after learning about the pregnancy and provided neither emotional nor financial support. The forensic records did not contain reliable information regarding contraceptive use, recognition of fetal movements, prenatal care, or her subjective emotional experience throughout pregnancy.

In her account of the offense, she stated:

“I was seven months pregnant, and when I told the child’s father, I said I didn’t want anything from him, I just wanted him to acknowledge the child, but he refused. He started to assault me. One day, I began to feel ill. I had hidden the pregnancy. I passed out, and when I woke up, the baby was inside a backpack. I took a motorcycle taxi to hide the bag with the baby because I was afraid of being arrested.”

The delivery reportedly occurred unexpectedly and without medical assistance. She described losing consciousness during the event and awakening with the newborn already inside a backpack. Immediately afterward, she reported acting in a confused and fearful manner, attempting to conceal the body because she feared criminal prosecution. During the forensic interview, she expressed distress when recalling these events and became tearful on several occasions.

Following the index offense, she established a stable relationship and had two additional children from different relationships. At the time of the forensic evaluation, she had been living with her partner before incarceration. She had previously worked as both a housemaid and a supermarket cashier but was unemployed.

At the psychiatric examination, the examinee presented with adequate hygiene and grooming. She was cooperative and answered questions promptly. She was alert, attentive, lucid, fully oriented, and demonstrated preserved recent and remote memory. Speech was spontaneous, coherent, and goal directed. Thought processes were logical and organized, with no evidence of formal thought disorder, hallucinations, or delusional ideation. Her intellectual functioning appeared compatible with her educational level and sociocultural background. She successfully performed simple calculations and interpreted proverbs appropriately. Her mood was anxious, affect was emotionally labile, and she cried while recounting the pregnancy and the events surrounding the delivery.

### 3.2 Case 2

B., a 21-year-old single white woman lived with family members. She was charged with killing her two-year-old son by repeatedly striking him with a sharp instrument.

According to the forensic records, she had a longstanding history of severe psychiatric illness beginning before the offense, with multiple psychiatric hospitalizations due to episodes of psychomotor agitation, aggressive behavior, and psychotic symptoms. Family members reported progressive behavioral deterioration over several years, including episodes of aggression directed toward relatives. At one point, she reportedly attempted to kill her older brother during a psychotic episode.

Although she had previously received inpatient psychiatric treatment, she was no longer engaged in regular outpatient psychiatric follow-up before the offense. Information regarding the reasons for treatment discontinuation was unavailable in the forensic records. Likewise, no information was available regarding psychiatric monitoring during pediatric follow-up of the child or referrals to child protection services before the homicide.

There was no documented history of alcohol or illicit substance use. Family members consistently associated her episodes of violence with exacerbations of psychiatric illness rather than substance intoxication.

During the psychiatric evaluation, she was minimally cooperative, responding to most questions with “I don’t know anything” while maintaining a markedly guarded and dissimulative attitude. She appeared suspicious throughout the interview. When questioned about perceptual disturbances, she admitted hearing voices, stating:

“I hear them—the spirits want to kill me.”

She further reported that these voices instructed her and warned that her son was in imminent danger. She expressed persecutory delusions, believing that unknown individuals and supernatural forces were monitoring her and plotting against her. At times, she suggested that killing her son was the only way to protect him from a fate worse than death, revealing delusional beliefs compatible with altruistic or persecutory psychotic ideation.

Her thought processes were markedly disorganized, with tangential, fragmented, and occasionally incoherent responses. She displayed inappropriate laughter, blunted affect, markedly impaired volition, and poor goal-directed behavior. Reality testing was severely compromised.

Based on the forensic psychiatric evaluation and the available longitudinal clinical history, the examinee met diagnostic criteria for schizophrenia (ICD-10 F20.0 (22)/ICD-11 6A20 (23)), characterized by persistent persecutory delusions, auditory hallucinations, severe thought disorganization, and profound impairment of judgment and behavioral control. The psychotic symptoms were judged to be directly related to the offense and were considered to have substantially impaired her capacity for understanding reality and determining her conduct at the time of the homicide.

## 4 Discussion

In Brazil, the criteria adopted by the Penal Code for assessing criminal responsibility is the biopsychological model: responsibility is only excluded if, at the time of the crime, the agent, due to mental illness or intellectual disability, was incapable of understanding (awareness of the illicit nature of the act) and self-determination (freedom to choose whether or not to commit the crime) (21). The biopsychological model requires verifying the existence of a causal link between the abnormal mental state and the criminal act, meaning that this state, contemporaneous with the act, must have completely or partially deprived the agent of either cognitive or volitional capacities. A forensic psychiatric examination is essential whenever there are doubts about the accused’s mental sanity.

Case 1 illustrates an important limitation inherent in retrospective forensic psychiatric assessment. Although the examinee did not present evidence of psychosis, major mood disorder, intellectual disability, or significant cognitive impairment at the time of the forensic examination, her mental status at that later assessment cannot be assumed to represent her mental state at the time of childbirth and the alleged offense. The legally relevant question is not whether psychiatric abnormalities were present during the forensic interview, but whether a mental disorder or transient alteration of mental functioning was present at the time of the offense and, if so, whether it substantially impaired her capacity for understanding or self-determination.

This distinction is particularly relevant because the examinee described an unexpected and unattended delivery and reported losing consciousness, subsequently awakening to find the newborn inside a backpack. This account is difficult to interpret retrospectively. In the absence of contemporaneous medical examination or sufficiently detailed obstetric documentation, several alternative explanations must be considered, including physiological loss or alteration of consciousness associated with childbirth, acute confusion, dissociative phenomena, severe emotional distress, a brief psychotic state, impaired or fragmented recall, and inaccurate retrospective reporting. Deliberate fabrication or malingering represents another possible explanation, but cannot be inferred solely from the unusual or uncorroborated nature of the account.

The available evidence did not establish a history of psychiatric disorder, previous psychiatric treatment, suicidality, substance use, or neurological illness. Her later forensic examination showed preserved orientation, memory, thought organization, reality testing, and intellectual functioning, without hallucinations or delusions. However, these findings primarily describe her condition at the time of examination and have limited ability to exclude a brief psychiatric, dissociative, confusional, or physiological alteration occurring specifically during childbirth.

Her behavior surrounding the event also requires cautious interpretation. Pregnancy concealment and subsequent concealment of the newborn’s body may be consistent with awareness of the circumstances and fear of legal consequences, but these behaviors are not, in isolation, definitive evidence of intact mental functioning throughout the entire event. Pregnancy concealment itself should not automatically be equated with premeditation. It may occur in the context of fear, shame, psychosocial adversity, maladaptive coping, or psychological processes related to pregnancy denial or dissociation. In the present case, the examinee acknowledged having concealed a pregnancy that she knew existed, which conceptually favors pregnancy concealment rather than complete pregnancy denial.

Sablone et al. (24) provide a particularly relevant framework for interpreting Case 1. The authors emphasize that denial of pregnancy is a heterogeneous phenomenon involving different degrees of cognitive and affective awareness and that its relationship with neonaticide cannot be explained solely by the degree to which the pregnancy is consciously recognized. In their case, two pregnancies in the same woman were characterized by different levels of awareness and markedly different outcomes: a pervasive denial culminated in adoption, whereas a subsequent pregnancy characterized by greater, although fluctuating, awareness ended in neonaticide. This observation suggests that pregnancy awareness alone may be insufficient to predict neonatal outcome and that circumstances surrounding labor and delivery, coping abilities, availability of social support, and access to healthcare may be equally important (24).

Nevertheless, given the examinee’s reported fear of family and social reactions, paternal rejection, and intimate-partner violence, Sablone et al. (24)’s framework is particularly informative: the authors describe childbirth following pregnancy denial as a potentially acute psychological crisis in which panic, confusion, dissociative symptoms, intermittent amnesia, and transient alterations in consciousness may occur. This framework is relevant to the examinee’s report of an unexpected unattended delivery, loss of consciousness, fragmented recall, and confused behavior immediately afterward, although the retrospective nature of the available evidence does not allow these phenomena to be established diagnostically. Thus, concealment of pregnancy and possible transient peripartum psychopathology should be considered separately when reconstructing her mental state at the time of the alleged offense.

Accordingly, the forensic conclusion in Case 1 should be understood as a retrospective judgment based on the totality of the available evidence rather than as proof that no transient alteration of mental state could have occurred. The available records did not provide sufficient corroborating evidence to establish that a psychotic, dissociative, confusional, or other pathological mental state was present at the legally relevant time or that such a state impaired her cognitive or volitional capacities to the degree required for exclusion or reduction of criminal responsibility. This distinction—between absence of evidence sufficient to establish forensic impairment and evidence demonstrating that no transient alteration occurred—is essential when interpreting the case.

More generally, retrospective evaluations of criminal responsibility in filicide are vulnerable to several sources of uncertainty. The evaluator is reconstructing a mental state that may no longer be directly observable and must often rely on retrospective self-report, incomplete medical and legal records, collateral accounts, and behavior before and after the offense. Hindsight bias may encourage interpretation of earlier behavior in light of the known outcome; confirmation bias may favor information consistent with an initial diagnostic hypothesis; and post-offense concealment or apparently organized behavior may be overinterpreted as demonstrating preserved cognitive and volitional capacities throughout the offense. Conversely, the severity of the outcome itself should not be taken as evidence that a severe mental disorder must have been present.

Forensic conclusions should therefore be based on convergence among longitudinal psychiatric history, contemporaneous evidence, collateral information, characteristics of the offense, symptom chronology, and the relationship between any identified psychopathology and the legally relevant cognitive and volitional capacities. When contemporaneous evidence is limited, as in Case 1, the degree of forensic certainty should be explicitly qualified.

In Case 2, the forensic psychiatric evaluation revealed that, at the time of the offense, the examinee exhibited psychotic symptoms, including auditory hallucinations commanding her and persecutory delusional ideation. She was considered criminally irresponsible due to the existence of a mental illness, diagnosed as schizophrenia, and was referred for psychiatric treatment in a forensic facility in the state of Rio de Janeiro, to receive psychiatric treatment (antipsychotic medication and psychotherapy).

Empirical studies have found that multiple factors are associated with filicide (25). They suggest that among most women who commit filicide, a set of stressors leads to the act. Motivating factors include financial difficulties, social isolation, single motherhood, employment problems, domestic issues, upbringing and maternal history, childhood sexual abuse, marital problems, jealousy, alcohol abuse, physical illness, mood disorders, or psychotic symptoms.

Parental risk factors for filicide include (26): acute psychiatric symptoms, as in case 2; dissociative states during the puerperal period; antisocial or borderline personality disorders or traits; substance abuse; poor coping skills; feelings of being overwhelmed; a history of domestic violence and childhood abuse; and chronic child maltreatment. Risk factors in children include physical or mental disabilities, low birth weight, prematurity, and persistent crying, all of which increase the likelihood of infanticide by a parent.

In a case-control study published in 2025, Bramante and Di Florio (27) examined cases of neonaticide and infanticide from a forensic psychiatric perspective and concluded that a prior psychiatric history—particularly affective and psychotic disorders—was present in a significant proportion of perpetrators, as in case 2. The author emphasizes the importance of distinguishing psychopathological factors from predominantly social factors, as this distinction directly impacts forensic interpretation regarding criminal responsibility.

Although prospective studies are considered methodologically superior for establishing risk factors, the relative rarity of filicide makes them difficult to conduct. Future studies should investigate various risk factors for filicide, including depression, sociodemographic factors, sociocultural environment, prior psychiatric history, and family conflict or violence.

An important point to emphasize is that denial of pregnancy (failing to acknowledge one’s own pregnancy) and active concealment (hiding the pregnancy due to fear or shame) are commonly observed in cases of neonaticide (13, 24). Women who commit neonaticide frequently report denial or concealment of pregnancy out of fear or shame, which aligns with the dynamics observed in the first case presented in this study. In Case 1, for instance, the woman concealed her pregnancy — a behavior that is widely described in the literature internationally, reinforcing the patterns seen in these cases.

The review by Ssekitto et al. (2024) (4) identified unwanted pregnancy as one of the factors most consistently associated with filicide across the included African studies. Cases of neonaticide were frequently linked to concealed or unrecognized pregnancies that continued until delivery. Evidence from South Africa suggested that these events may occur even where abortion and contraceptive services are legally available, pointing to broader gaps in reproductive health care and social support. The review also noted that paternal denial, limited involvement of fathers, and the absence of social recognition of the pregnancy may contribute to circumstances surrounding neonatal abandonment and death.

Barnes (28) distinguishes pregnancy denial, in which the woman is unaware of the pregnancy, from pregnancy concealment, in which the pregnancy is recognized but hidden from others. Although conceptually distinct, both may represent maladaptive responses to overwhelming psychological distress and are frequently associated with childhood trauma and dissociative processes. Accordingly, concealment should not be interpreted as evidence of premeditation, but rather as a possible manifestation of psychological disorganization and difficulty integrating the reality of the pregnancy. Barnes (28) also emphasizes the forensic implications of pregnancy denial, arguing that its absence from the Diagnostic and Statistical Manual of Mental Disorders (DSM) (29) may hinder its recognition in forensic psychiatric evaluations. Greater diagnostic recognition could therefore improve both clinical assessment and judicial interpretation of neonaticide cases.

Psychiatrists, obstetricians, and pediatricians should evaluate postpartum women for clinically significant psychiatric symptoms (14). Women presenting with severe depression or psychosis require prompt assessment by mental health services. When thoughts of harm involving the infant are reported, clinicians should distinguish common intrusive thoughts from genuine homicidal ideation. Intrusive thoughts of infant harm may occur during the perinatal period and are typically unwanted, distressing, and ego-dystonic. In the absence of intent, planning, psychotic symptoms, or impaired reality testing, they should not be interpreted as evidence that the mother is likely to harm her child. In contrast, thoughts of harming the infant associated with intent or planning, command hallucinations, delusional beliefs involving the child, or severe depression with suicidal or altruistic homicidal thinking warrant immediate and comprehensive risk assessment. Psychiatric hospitalization should be considered when severe depression or psychosis is associated with a significant risk of harm to the mother or the infant.

Although neonaticide, infanticide, and filicide have been extensively described in the psychiatric literature, the present report contributes to the field by illustrating two distinct psychopathological pathways leading to child homicide within the Brazilian forensic context. The first case represents a concealed pregnancy associated with significant psychosocial adversity but without evidence of a major psychiatric disorder or impairment of criminal responsibility. In contrast, the second case illustrates filicide committed during an acute psychotic episode characterized by persecutory delusions, auditory hallucinations, and profound impairment of reality testing.

An additional contribution of this study concerns the Brazilian legal framework. Unlike Infanticide Acts adopted in several common-law countries, Article 123 of the Brazilian Penal Code (30) requires that the homicide occur under the influence of the *puerperal state*, a legal concept that has no direct diagnostic equivalent in either the DSM-5 (29) or the ICD-11 (23). Consequently, Brazilian forensic psychiatrists must distinguish between transient psychological phenomena occurring during the puerperium and severe psychiatric disorders, such as schizophrenia or psychotic depression, which have independent forensic implications regarding criminal responsibility. These cases demonstrate how similar criminal outcomes may result from fundamentally different psychopathological mechanisms, requiring different forensic conclusions, legal interpretations, and preventive strategies.

The present cases also emphasize the importance of strengthening perinatal mental health services. Although filicide is a rare event, numerous studies indicate that severe psychiatric disorders during pregnancy and the postpartum period are among the strongest potentially modifiable risk factors. Early identification and continuous treatment of women with psychotic disorders, bipolar disorder, severe depressive episodes, or previous postpartum psychosis should therefore constitute a public health priority.

The second case illustrates the consequences of discontinuity of psychiatric care in a woman with recurrent psychotic illness. Regular psychiatric follow-up, prompt management of treatment discontinuation, family psychoeducation, and coordinated care involving psychiatrists, obstetricians, pediatricians, primary care physicians, psychologists, and social workers may reduce the likelihood of severe psychiatric decompensation and improve maternal and child safety. Likewise, socially vulnerable women experiencing unwanted or concealed pregnancies should receive accessible psychosocial support and appropriate reproductive healthcare, even in the absence of a diagnosable psychiatric disorder.

Homicidal ideation occurring in the context of psychotic disorders differs fundamentally from intrusive thoughts. In psychosis, thoughts of harming the child may arise from persecutory or altruistic delusions, command auditory hallucinations, or profound disturbances in reality testing. These experiences are often ego-syntonic or perceived as justified by the patient and may significantly increase the risk of violent behavior if left untreated.

Accordingly, screening during the perinatal period should not focus solely on the presence of thoughts about harming the infant but rather on their psychopathological context. Assessment should include the mother’s level of insight, the presence of psychotic symptoms, emotional response to the thoughts, degree of reality testing, behavioral intent, and associated psychiatric disorders. This distinction is essential to avoid stigmatizing mothers who experience common intrusive thoughts while ensuring that women with severe mental illness receive urgent psychiatric evaluation and appropriate intervention.

Despite statistical and empirical evidence suggesting a positive relationship between severe mental disorders and violent behavior, these cases represent only a small proportion of overall community violence. In countries with high rates of violence, such as Brazil — where violence and crime are closely tied to poor socioeconomic conditions — the proportion of homicides associated with mental illness may be even lower.

The goal of studying the association between violence and mental disorders is not to stigmatize but to better understand contributing factors and propose mental health policies and therapeutic interventions for patients with mental disorders and violent behavior. The literature also highlights that many of these women expressed deep regret for not seeking prior help, which underscores the importance of a preventive approach and careful psychiatric assessments during the perinatal period (13).

## 5 Conclusion

Psychiatric patients with a criminal history would benefit from treatment programs that often exclude them. The presence of a criminal record should indicate a greater need for integrated approaches, not grounds for treatment exclusion. Mental health services must work to prevent loss of contact and treatment nonadherence, which often precede violent behavior in individuals with severe mental disorders.

Additionally, it is crucial for society and government authorities to reduce barriers to psychiatric and psychosocial treatment access. Supporting pregnant women without mental disorders but in conditions of social vulnerability is also important. Psychiatrists, obstetricians, and pediatricians must screen postpartum women for psychiatric symptoms to help reduce these tragic events.

Future studies should continue investigating various risk factors for maternal filicide, such as depression, psychosis, sociodemographic and sociocultural factors, prior psychiatric history, and family conflict or violence. Studying the motivational factors behind filicidal behavior can provide insights for developing therapeutic interventions for women with mental disorders at risk of this and other violent behaviors.

The present study has several strengths. First, it provides detailed forensic psychiatric descriptions of two uncommon but clinically important forms of maternal child homicide evaluated within the Brazilian criminal justice system. Second, it compares two distinct psychopathological pathways leading to child homicide—one associated predominantly with psychosocial vulnerability and concealed pregnancy, and the other with severe psychotic illness—highlighting their different forensic and legal implications. Third, the study discusses these cases within the context of the Brazilian legal concept of the *puerperal state*, thereby contributing to the international literature by describing a medico-legal framework that differs from those adopted in many other countries.

This study also has important limitations. It is based on only two retrospective forensic cases, which limits the generalizability of the findings. Some clinical and psychosocial information, including detailed obstetric history, prenatal care, family dynamics, reasons for treatment discontinuation, and child protection records, was unavailable because these variables had not been systematically documented in the original forensic records. Consequently, causal inferences cannot be established, and the findings should be interpreted as illustrative rather than representative. Future multicenter studies with larger samples and prospective designs are needed to better characterize the relationship between psychopathology, psychosocial vulnerability, and maternal child homicide.

## Data Availability

The original contributions presented in the study are included in the article/supplementary material, further inquiries can be directed to the corresponding author/s.
